# Three-Dimensional Analysis of Orbital Sexual Dimorphism in a Romanian Sample: Implications for Sex Estimation and Forensic Facial Reconstruction

**DOI:** 10.7759/cureus.117618

**Published:** 2026-10-07

**Authors:** Mihai Sabin Scarlat, George Cristian Curca

**Affiliations:** 1 Department of Legal Medicine and Bioethics, Faculty of Medicine, Carol Davila University of Medicine and Pharmacy, Bucharest, ROU; 2 Department of Forensic Pathology and Clinical Legal Medicine, ”Mina Minovici” National Institute of Legal Medicine, Bucharest, ROU

**Keywords:** forensic anthropology, geometric morphometrics, human identification, sex estimation, sexual dimorphism

## Abstract

Introduction

The orbit exhibits sexual dimorphism and may provide useful information for sex estimation in forensic anthropology. However, data comparing orbital measurements with geometric morphometrics are limited for the contemporary Romanian population. This study evaluated the sexual dimorphism of the orbital region using both approaches and assessed their performance for sex estimation, with potential indirect relevance to forensic facial reconstruction.

Methods

This study included 163 CT head scans from contemporary Romanian adults, including 78 (47.9%) males and 85 (52.1%) females. Ten anatomical landmarks were digitized in three-dimensional space, and their Cartesian coordinates were used for both conventional linear measurements and geometric morphometric analysis. Six conventional orbital measurements were evaluated from three-dimensional anatomical landmarks using univariate and multivariate discriminant function analyses with leave-one-out cross-validation (LOOCV). Geometric morphometric analysis was performed using generalized Procrustes analysis and principal component analysis. Sexual dimorphism in centroid size and Procrustes shape was assessed, and allometry was investigated through multivariate regression of shape on log centroid size, including pooled within-sex and sex-specific analyses. Discriminant analysis was also performed before and after allometric correction.

Results

Orbital breadth, interorbital breadth, and biorbital breadth were significantly greater in males, whereas orbital height showed limited sexual dimorphism. Biorbital breadth was the most accurate individual conventional measurement, correctly classifying 119/163 (73.0%) individuals after cross-validation, whereas the multivariate linear model correctly classified 114/163 (69.9%) individuals. Significant sex-related differences were also identified in orbital shape and centroid size. Uncorrected Procrustes shape correctly classified 116/163 individuals (71.2%) after cross-validation, increasing to 121/163 (74.2%) after correction for allometry. Centroid size alone correctly classified 119/163 (73.0%) individuals, while the combined uncorrected shape and centroid size model achieved the highest cross-validated accuracy, correctly classifying 125/163 (76.7%) individuals, including 67/85 (78.8%) females and 58/78 (74.4%) males.

Conclusion

Conventional linear measurements showed lower cross-validated classification accuracy than the combined centroid size and shape model. Integrating three-dimensional orbital size and shape may therefore provide complementary information that may improve sex estimation with potential indirect relevance to forensic facial reconstruction.

## Introduction

Sex estimation is a component of the biological profile and an important step in evaluating unknown human remains. Reliable sex estimation can narrow the pool of potential identities by half. The pelvis generally exhibits the greatest degree of skeletal sexual dimorphism and is therefore considered the most reliable for sex estimation [1]. However, the pelvis is not always recovered or sufficiently preserved for analysis. In some forensic scenarios, the skull may represent the only available skeletal element and can therefore provide valuable information for sex estimation, although the resulting accuracy depends on the anatomical region examined and the methodological approach employed [2].

The skull is not only valuable for the biological profile [2-5], but it also forms the anatomical basis for forensic facial reconstruction. If there is no antemortem information available, facial reconstruction may assist the investigation by generating a representation of the probable facial appearance, potentially facilitating recognition by relatives or other persons familiar with the individual and providing new investigative leads [6]. The accuracy of such an approximation depends, in part, on accurate information about the biological profile. Sex is especially relevant in this context because it influences the reconstructed facial appearance [7]. Consequently, accurate sex estimation has a dual role: it narrows the pool of potential identities and provides biologically relevant information for the subsequent reconstruction of the face.

The orbit exhibits sexual dimorphism [8]. However, the degree of these differences varies among populations owing to genetic, developmental, and environmental influences [9]. Consequently, population-specific studies are necessary not only to characterize sex-related orbital variation but also to determine its practical value for sex classification. Previous studies have reported variability in classification performance, with accuracy based on linear orbital measurements ranging from 48% to 83% [10,11], whereas shape-based approaches using geometric morphometrics have achieved accuracies ranging from 68.1% to 80% [12,13]. This variability shows that orbital sexual dimorphism is not universally expressed and that both population background and methodological approach must be considered when using sex-estimation models.

Studies investigating sexual dimorphism of the orbit and its performance for sex estimation are limited in the Romanian population. To our knowledge, only one study [14] has included orbital measurements in sex-estimation analyses, and no previous study has used geometric morphometrics on the orbit or assessed its discriminatory performance for sex classification in a contemporary Romanian adult sample.

The primary objectives of this study were to assess three-dimensional (3D) sexual dimorphism of the orbit in a contemporary Romanian sample and to evaluate its utility for population-specific sex estimation. Secondary objectives were to compare conventional linear morphometry with geometric morphometric approaches and to assess the internally validated classification performance of the resulting sex-estimation models using leave-one-out cross-validation (LOOCV).

## Materials and methods

Study design and sample

In this retrospective cross-sectional study, medico-legal case files from the entire year 2024 were screened for the presence of head CT scans. The inclusion criteria were age ≥18 years, complete visualization of both orbital regions, and image quality sufficient for 3D reconstruction. Because the CT examinations had been acquired using different scanners and imaging protocols, technical criteria were applied to improve data uniformity. Accordingly, only examinations with a slice thickness ≤1.5 mm, a 512 × 512 pixel matrix, and an in-plane pixel spacing <1 mm were included. The exclusion criteria were fractures, deformities, and osseous pathology affecting the orbital region. After application of the inclusion and exclusion criteria, 163 medico-legal case files containing eligible head CT examinations remained for analysis. Information on sex was also obtained from the corresponding medico-legal case files, resulting in a final sample of 78 males and 85 females (Figure 1).

**Figure 1:**
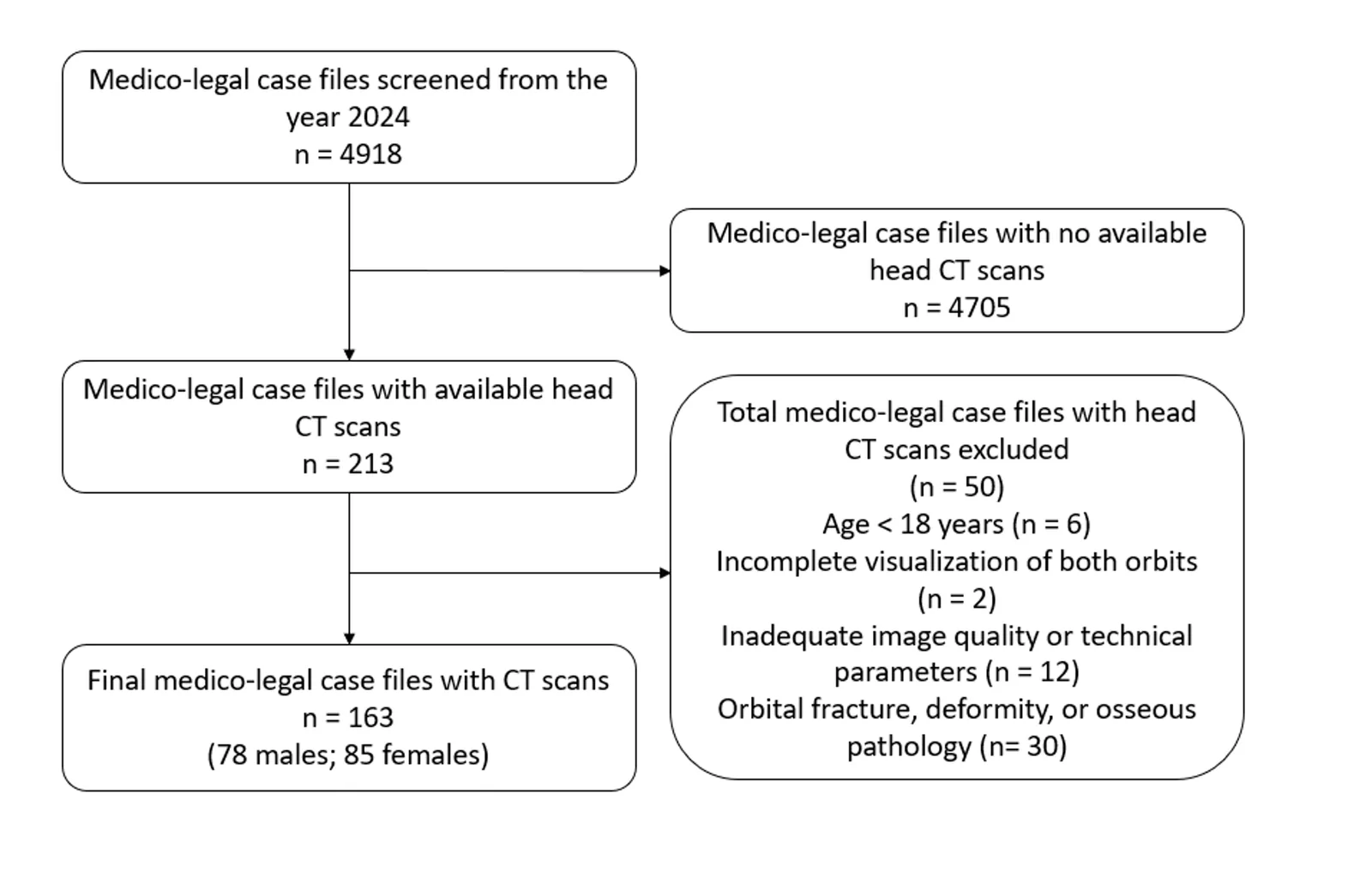
Flow diagram of medico-legal case file screening CT, computed tomography

No formal a priori sample-size calculation was performed, as the final sample comprised all eligible CT examinations available during the predefined study period after application of the inclusion and exclusion criteria. Sample adequacy was considered in relation to model complexity, with the number of observations exceeding the number of predictors in all multivariate models.

The study was approved by the Institutional Review Board (IRB) of the National Institute of Legal Medicine “Mina Minovici” (approval no. 8048/05.06.2026) and adhered to the Declaration of Helsinki. Due to the retrospective nature of the study, the IRB of the National Institute of Legal Medicine “Mina Minovici” waived the requirement for informed consent.

Landmark acquisition

The DICOM files retrieved from the medico-legal case files were imported into 3D Slicer (version 5.12.2; Slicer Community) [15], where the volume rendering module generated 3D reconstructions of the skull. The anatomical landmarks presented in Table 1 [16] were manually positioned on the surface of the 3D reconstruction according to their definitions.

**Table 1:** Definitions of anthropological landmarks

Landmark	Anatomical definition
Dacryon	Point on the frontal bone where the frontal, lacrimal, and maxillary sutures meet
Ectoconchion	The intersection of the most anterior surface of the lateral border of the orbit with an imaginary line bisecting the orbit along its long axis
Upper orbital border	The point on the upper orbital border intersected by an imaginary line drawn perpendicular to the long axis of the orbit and bisecting it.
Lower orbital border	The point on the lower orbital border intersected by an imaginary line drawn perpendicular to the long axis of the orbit and bisecting it
Frontomalare anterior	Most anterior point on the frontozygomatic suture

Five landmarks were placed on each orbit, resulting in a total of 10 landmarks per CT scan (Figure 2).

**Figure 2:**
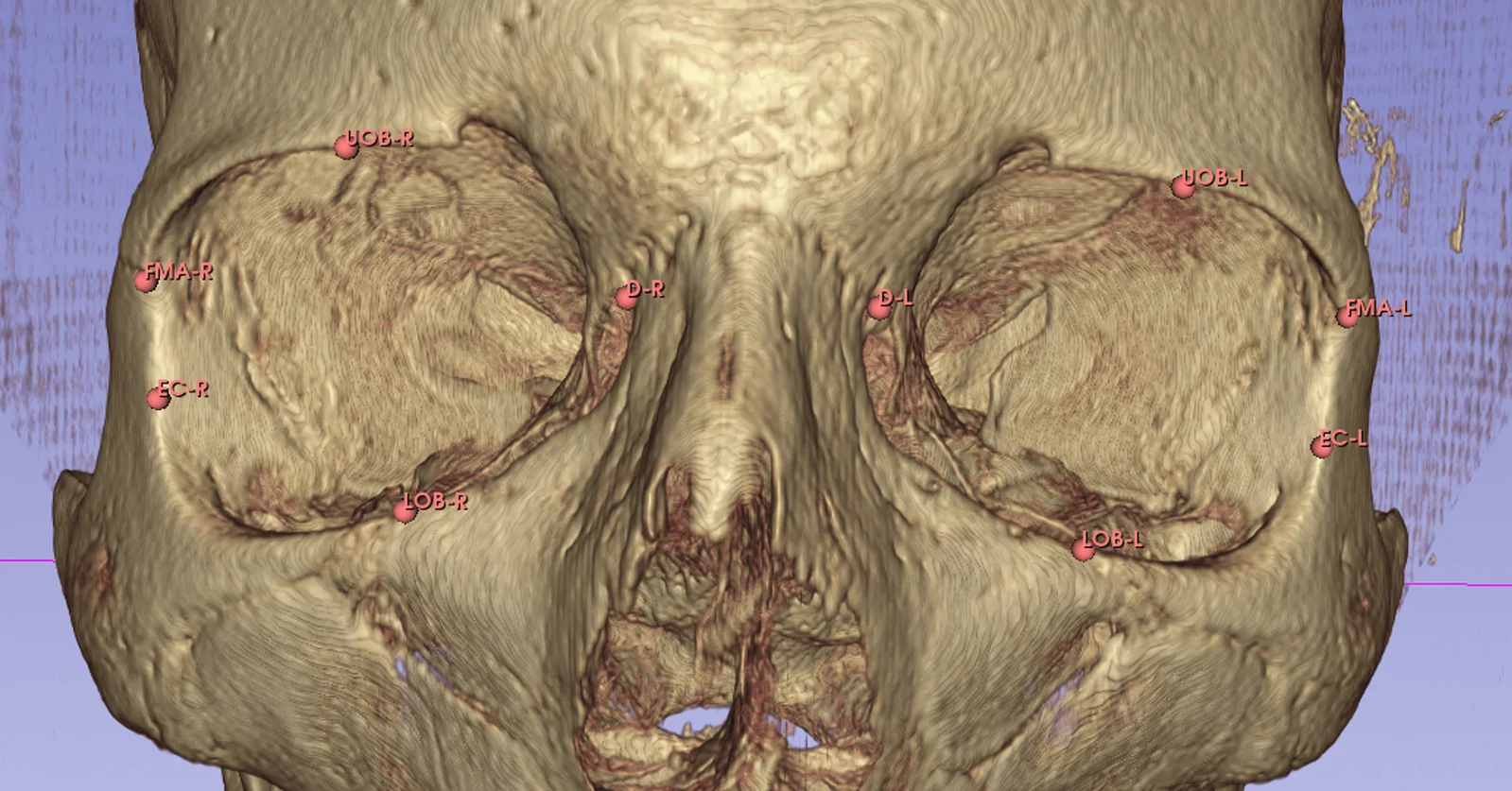
A 3D reconstruction of a CT scan showing landmark positions Volume rendering model in 3D Slicer version 5.12.2. D, Dacryon; EC, ectoconchion; UOB, upper orbital border; LOB, lower orbital border; FMA, frontomalare anterior; R, right; L, left

The Cartesian coordinates (X, Y, Z) of each landmark were exported and used to derive the linear measurements and for geometric morphometric analysis; linear measurements were calculated directly from the 3D Cartesian coordinates as Euclidean distances, and for any two landmarks with coordinates (x₁, y₁, z₁) and (x₂, y₂, z₂), the three-dimensional distance was calculated as d = √[(x₂ − x₁)² + (y₂ − y₁)² + (z₂ − z₁)²].

The linear measurements were as follows: orbital breadth (OBB), the distance between the dacryon and ectoconchion; orbital height (OBH), the distance between the superior and inferior orbital border along an imaginary line perpendicular to the long axis of the orbit and bisecting it; interorbital breadth (IOB), the distance between the right and left dacryon; biorbital breadth (BOB), the distance between the right and left ectoconchion [16]. The orbital index (OI) was calculated separately for each side as OBH divided by OBB × 100.

Assessments of reproducibility

Intra-observer reproducibility was assessed in a random subsample of 30 CT scans, and sex information was not provided to the observer during the reproducibility assessment. The selected scans were independently re-landmarked by the same observer in separate sessions. For each landmark, 3D localization error was calculated as the Euclidean distance between the corresponding coordinate triplets obtained from the two sessions. Landmark-specific errors were expressed in millimeters and reported using the mean, standard deviation (SD), minimum, and maximum values.

Because the linear measurements were calculated from the landmark coordinates, differences in landmark placement between sessions could result in differences in the derived measurements [17,18]. Therefore, the derived linear orbital measurements were independently recalculated from the landmark coordinates obtained in each session, and their reproducibility was assessed using a two-way mixed-effects, absolute-agreement, single-measure intraclass correlation coefficient (ICC(2,1)), with 95% confidence intervals to determine whether intra-observer variation in landmark placement affected the final linear values used in the statistical analyses.

Statistical analysis

Statistical analyses of the linear measurements were performed using jamovi (version 2.7; The jamovi project) [19]. Continuous variables were summarized as mean ± SD. Sex differences in age and orbital measurements were assessed using independent-samples t-tests, and Welch’s t-test was applied when the assumption of homogeneity of variances was violated. Bilateral differences were evaluated using paired-samples t-tests. Effect sizes were expressed as Cohen’s d. The sexual dimorphism index (SDI) was calculated as the ratio of male-to-female mean (M/F). Statistical significance was set at p < 0.05.

To assess the ability of linear orbital measurements to estimate sex, univariate discriminant function analyses were performed for each measurement separately, whereas the six linear measurements were used for multivariate discriminant function analysis. Overall and sex-specific accuracies were calculated using LOOCV, with equal prior probabilities assigned to females and males. For the multivariate model, the structure coefficients and standardized canonical discriminant coefficients were examined to assess the contribution of each variable to the discriminant function.

Geometric morphometric analyses were conducted using MorphoJ (version 1.08.02; Christian P. Klingenberg) [20]. The 3D landmark coordinates were subjected to generalized Procrustes analysis (GPA). Principal component analysis (PCA) of the Procrustes coordinates was used to characterize orbital shape variation. Sex differences in centroid size were assessed using an independent-samples t-test, while shape differences were evaluated by discriminant function analysis performed on the Procrustes coordinates with permutation testing and LOOCV. Allometry was assessed by multivariate regression of Procrustes coordinates on log centroid size, including pooled within-sex and sex-specific regressions. Discriminant function analysis was repeated on allometry-adjusted residuals that were obtained from the pooled within-sex regression of the complete sample before discriminant classification and LOOCV. Additional discriminant analysis models were performed in RStudio (version 4.6.0; R Foundation for Statistical Computing, Vienna, Austria) [21] to evaluate sex estimation performance based on centroid size alone and the combination of shape and centroid size. For the combined model, shape was represented by all principal component scores derived from the Procrustes coordinates, and centroid size was included as an additional predictor. Classification performance was evaluated using LOOCV, with equal prior probabilities assigned to females and males.

## Results

Data from 163 head CT scans, comprising 78 males (47.9%) and 85 females (52.1%), were included. The average age was 42.65 ± 14.67 years for males and 45.02 ± 16.24 years for females. No statistically significant difference in age was observed between the sexes (t = −0.97, p = 0.331), indicating comparable mean ages between the groups.

Intra-observer assessment showed mean 3D landmark localization errors ranging from 0.351 ± 0.185 mm to 0.500 ± 0.220 mm, with all individual errors below 1 mm (Table 2).

**Table 2:** Intra-observer landmark localization error Data are presented as mean ± standard deviation and range (minimum-maximum) in millimeters (mm).

Landmark	Mean error ± SD (mm)	Range (mm)
Dacryon right	0.470 ± 0.182	0.178–0.796
Ectoconchion right	0.389 ± 0.171	0.129–0.743
Upper orbital border right	0.500 ± 0.220	0.207–0.899
Lower orbital border right	0.409 ± 0.217	0.084–0.954
Frontomalare anterior right	0.403 ± 0.178	0.108–0.792
Dacryon left	0.438 ± 0.225	0.107–0.894
Ectoconchion left	0.448 ± 0.212	0.072–0.858
Upper orbital border left	0.436 ± 0.195	0.071–0.878
Lower orbital border left	0.437 ± 0.231	0.131–0.876
Frontomalare anterior left	0.351 ± 0.185	0.079–0.901

The derived orbital measurements showed ICC values ranging from 0.978 to 0.997 (Table 3).

**Table 3:** Intra-observer reliability of linear measurements Intra-observer reliability of linear measurements was assessed using a two-way mixed-effects, absolute-agreement, single-measure  [ICC(2,1)], with 95% confidence intervals. OBB, orbital breadth; OBH, orbital height; IOB, interorbital breadth; BOB, biorbital breadth; OI, orbital index; ICC, intraclass correlation coefficient; CI, confidence interval

Linear orbital measurement	ICC	95% CI
OBB right	0.991	0.983–0.995
OBB left	0.978	0.958–0.989
OBH right	0.981	0.965–0.990
OBH left	0.986	0.973–0.992
IOB	0.983	0.966–0.991
BOB	0.997	0.994–0.998
OI right	0.981	0.965–0.990
OI left	0.98	0.963–0.989

Overall, the observed localization errors and high ICC values indicated good intra-observer consistency and supported the reliability of the measurements.

Linear measurements results

Significant sex differences were observed for bilateral OBB, IOB, and BOB, with higher mean values in males (all p < 0.001). The largest effect size was observed for BOB (Cohen’s d = 0.993), followed by right (d = 0.958) and left (d = 0.917) OBB. The SDI was highest for IOB (SDI = 1.061), followed by right and left OBB (1.048 and 1.043, respectively) and BOB (1.037). In contrast, OBH did not differ significantly between sexes on either side (right: p = 0.193; left: p = 0.211) and showed only minimal proportional dimorphism (SDI = 1.012-1.015). Orbital indices were significantly higher in females for both the right (p = 0.003, d = 0.470) and left orbit (p = 0.009, d = 0.416), with corresponding SDI values below unity (0.967 and 0.971, respectively) (Table 4).

**Table 4:** Comparison of orbital dimensions between males and females Data are presented as mean ± standard deviation and range (minimum–maximum) in millimeters (mm). Sex differences were assessed using independent-samples t-tests. Student’s t-test was used when the assumption of equal variances was met; Welch’s t-test was used for OBB left because Levene’s test indicated unequal variances (*). A p-value of <0.05 was considered statistically significant. SDI, sexual dimorphism index; OBB, orbital breadth; OBH, orbital height; IOB, interorbital breadth; BOB, biorbital breadth; OI, orbital index

Variable	Males, mean ± SD	Range	Females, mean ± SD	Range	t (df)	p	Cohen’s d	SDI
OBB right	41.8 ± 2.12	37.2–46.7	39.9 ± 1.81	35.8–45.5	−6.11 (161)	<0.001	0.958	1.048
OBB left	41.7 ± 2.08	36.2–46.7	40.0 ± 1.58	35.2–44.6	−5.81 (143.12)*	<0.001	0.917	1.043
OBH right	33.2 ± 2.16	28.5–38.8	32.8 ± 2.08	28.2–38.8	−1.31 (161)	0.193	0.205	1.012
OBH left	33.7 ± 2.28	29.2–40.3	33.2 ± 2.07	28.2–39.0	−1.26 (161)	0.211	0.197	1.015
IOB	20.9 ± 2.04	15.1–25.7	19.7 ± 1.96	15.6–24.7	−3.60 (161)	<0.001	0.564	1.061
BOB	97.6 ± 3.61	86.5–106.2	94.1 ± 3.46	85.5–105.0	−6.34 (161)	<0.001	0.993	1.037
OI right	79.7 ± 5.73	64.7–91.9	82.4 ± 5.39	68.2–97.5	3.00 (161)	0.003	0.47	0.967
OI left	80.8 ± 5.63	67.7–94.5	83.2 ± 5.59	67.2–95.5	2.65 (161)	0.009	0.416	0.971

Bilateral Differences 

Bilateral comparisons showed no significant difference in OBB between the right and left sides (mean difference = −0.031 mm, 95% CI: −0.191 to 0.130, p = 0.706, Cohen’s d = −0.030). In contrast, OBH was significantly greater on the left side (mean difference = −0.424 mm, 95% CI: −0.556 to −0.292, p < 0.001, d = −0.498). The OI was also significantly higher on the left (mean difference = −0.956, 95% CI: −1.396 to −0.516, p < 0.001, d = −0.336) (Table 5).

**Table 5:** Bilateral differences in orbital measurements Data are presented as mean ± standard deviation, mean right-to-left difference (R−L), and 95% CI of the mean difference. Bilateral differences were assessed using paired-samples t-tests. OBB and OBH are expressed in millimeters (mm). A p-value of <0.05 was considered statistically significant. OBB, orbital breadth; OBH, orbital height; OI, orbital index; CI, confidence interval

Variable	Right, mean ± SD	Left, mean ± SD	Mean difference (R−L)	95% CI of mean difference	t (df)	p	Cohen’s d
OBB	40.8 ± 2.17	40.8 ± 2.02	−0.031	−0.191 to 0.130	−0.38 (162)	0.706	−0.030
OBH	33.0 ± 2.12	33.4 ± 2.18	−0.424	−0.556 to −0.292	−6.36 (162)	<0.001	−0.498
OI	81.1 ± 5.69	82.1 ± 5.71	−0.956	−1.396 to −0.516	−4.29 (162)	<0.001	−0.336

Sex-stratified analyses showed a consistent bilateral pattern in males and females. OBB did not differ significantly between sides in either males (mean right-left difference = 0.065 mm, p = 0.606, Cohen’s d = 0.059) or females (−0.119 mm, p = 0.256, d = −0.124). In contrast, OBH was significantly greater on the left in both males (mean difference = −0.421 mm, p < 0.001, d = −0.452) and females (−0.427 mm, p < 0.001, d = −0.550). The OI was likewise significantly higher on the left in males (mean difference = −1.102, p = 0.002, d = −0.366) and females (−0.822, p = 0.006, d = −0.305) (Table 6).

**Table 6:** Sex-specific bilateral differences in orbital measurements Data are presented as mean R−L differences and 95% CI of the mean difference. Sex-specific bilateral differences were assessed using paired-samples t-tests in males (n = 78; 47.9%) and females (n = 85; 52.1%). OBB and OBH differences are expressed in millimeters (mm). A p-value <0.05 was considered statistically significant. OBB, orbital breadth; OBH, orbital height; OI, orbital index; R−L, right-to-left; CI, confidence interval

Sex	Variable	Mean difference (R−L)	95% CI of mean difference	t (df)	p	Cohen’s d
Male	OBB	+0.065 mm	−0.186 to 0.317	0.517 (77)	0.606	0.059
Male	OBH	−0.421 mm	−0.631 to −0.211	−3.992 (77)	<0.001	−0.452
Male	OI	−1.102	−1.781 to −0.423	−3.233 (77)	0.002	−0.366
Female	OBB	−0.119 mm	−0.326 to 0.088	−1.143 (84)	0.256	−0.124
Female	OBH	−0.427 mm	−0.595 to −0.260	−5.069 (84)	<0.001	−0.550
Female	OI	−0.822	−1.403 to −0.241	−2.812 (84)	0.006	−0.305

Discriminant Function Analysis of Linear Measurements

Univariate discriminant analysis showed that BOB provided the strongest discrimination between sexes, with the highest eigenvalue (0.249), canonical correlation (0.447), and lowest Wilks’ λ (0.800). BOB achieved the highest overall classification accuracy, correctly classifying 119/163 individuals (73.0%). The bilateral OBB also showed useful discriminatory performance, with overall accuracies of 114/163 (69.9%) for the right side and 112/163 (68.7%) for the left side. IOB correctly classified 104/163 (63.8%) individuals, whereas the orbital indices showed lower classification performance, with 97/163 (59.5%) correctly classified for the right side and 100/163 (61.3%) for the left side. OBH showed no significant discriminatory ability and yielded chance-level classification rates with classification accuracies of 86/163 (52.8%) on the right and 87/163 (53.4%) on the left (Table 7).

**Table 7:** Univariate discriminant analysis of orbital measurements for sex classification Univariate discriminant function analyses performed separately for each orbital measurement. Statistical significance of each discriminant function was assessed using Wilks’ lambda and the corresponding chi-square test. Classification performance was evaluated using LOOCV and is presented as the number of correctly classified individuals/total number of individuals with the corresponding percentage [n/N (%)]. A p-value of <0.05 was considered statistically significant. OBB, orbital breadth; OBH, orbital height; IOB, interorbital breadth; BOB, biorbital breadth; OI, orbital index; LOOCV, leave-one-out cross-validation

Variable	Eigenvalue	Canonical correlation	Wilks’ λ	χ²	df	p	Male, n/N (%)	Female, n/N (%)	Overall, n/N (%)
OBB right	0.232	0.434	0.812	33.49	1	<0.001	54/78 (69.2%)	60/85 (70.6%)	114/163 (69.9%)
OBB left	0.215	0.421	0.823	31.24	1	<0.001	48/78 (61.5%)	64/85 (75.3%)	112/163 (68.7%)
OBH right	0.011	0.103	0.989	1.7	1	0.193	41/78 (52.6%)	45/85 (52.9%)	86/163 (52.8%)
OBH left	0.01	0.098	0.99	1.56	1	0.211	40/78 (51.3%)	47/85 (55.3%)	87/163 (53.4%)
IOB	0.08	0.273	0.926	12.41	1	<0.001	46/78 (59.0%)	58/85 (68.2%)	104/163 (63.8%)
BOB	0.249	0.447	0.8	35.72	1	<0.001	60/78 (76.9%)	59/85 (69.4%)	119/163 (73.0%)
OI right	0.056	0.23	0.947	8.73	1	0.003	46/78 (59.0%)	51/85 (60.0%)	97/163 (59.5%)
OI left	0.044	0.205	0.958	6.87	1	0.009	46/78 (59.0%)	54/85 (63.5%)	100/163 (61.3%)

The multivariate discriminant function based on the six conventional linear measurements showed a significant difference between males and females (eigenvalue = 0.332, canonical correlation = 0.500, Wilks’ λ = 0.750, χ²(6) = 45.35, p < 0.001). BOB showed the highest structure coefficient (0.866), followed by right (0.835) and left (0.804) OBB, indicating that breadth-related measurements had the strongest associations with the discriminant function (Table 8).

**Table 8:** Multivariate discriminant analysis of conventional orbital measurements Structure coefficients represent the correlations between each predictor and the discriminant function, whereas standardized coefficients indicate the relative contribution of each predictor after accounting for the other variables in the model. OBB, orbital breadth; OBH, orbital height; IOB, interorbital breadth; BOB, biorbital breadth

Variable	Structure coefficient	Standardized coefficient
OBB right	0.835	0.75
OBB left	0.804	0.413
OBH right	0.179	0.358
OBH left	0.172	−0.349
IOB	0.492	0.702
BOB	0.866	−0.356

Apparent classification correctly assigned 121/163 (74.2%) individuals, including 57/78 (73.1%) males and 64/85 (75.3%) females. Following LOOCV, 114/163 (69.9%) individuals were correctly classified, including 55/78 (70.5%) males and 59/85 (69.4%) females.

Geometric morphometric results

Following GPA, the superimposed landmark configurations illustrated the overall bilateral morphology of the orbital region and the inter-individual variation of the 10 3D landmarks across the 163 CT examinations (Figure 3).

**Figure 3:**
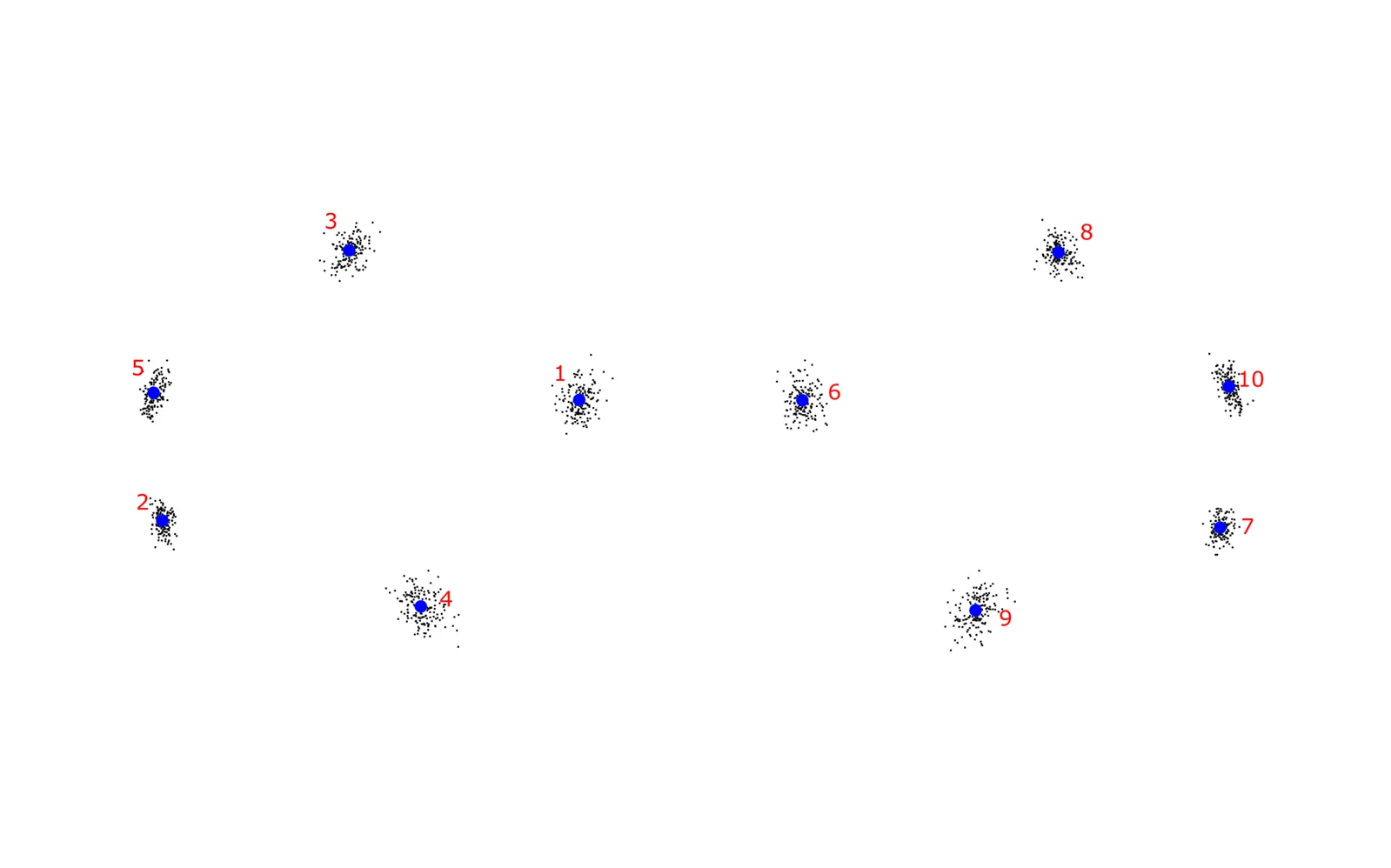
Generalized Procrustes analysis Procrustes coordinates are projected onto axes 1 and 3. Black points represent individual Procrustes-aligned landmark positions, whereas blue points indicate the mean landmark positions. Landmark numbers correspond to the following: 1, dacryon right; 2, ectoconchion right; 3, upper orbital border right; 4, lower orbital border right; 5, frontomalare anterior right; 6, dacryon left; 7, ectoconchion left; 8, upper orbital border left; 9, lower orbital border left; and 10, frontomalare anterior left.

Size

Centroid size differed significantly between sexes, with males showing larger orbital configurations than females. The mean centroid size was 122.40 ± 4.67 in males and 117.84 ± 4.36 in females. The difference was statistically significant, t(161) = −6.45, p < 0.001, with a large effect size (Cohen’s d = 1.01), indicating marked sexual dimorphism in overall orbital size.

Shape

PCA of the Procrustes-aligned coordinates identified 23 principal components that described orbital shape variation. PC1 accounted for 26.24% of the total variance, PC2 for 19.13%, and PC3 for 15.04%, with the first three components cumulatively explaining 60.42% of the total shape variation. The first five principal components accounted for 74.38% of the total variance (Table 9).

**Table 9:** Principal components with percentage of orbital shape variance Data are presented as the percentage of total orbital shape variance explained by each principal component and the corresponding cumulative percentage of variance. PC, principal component

Principal component	Percentage of variance (%)	Cumulative percentage of variance (%)
PC1	26.24	26.24
PC2	19.13	45.37
PC3	15.04	60.42
PC4	8.25	68.66
PC5	5.72	74.38

The distribution of individuals in the PC1 and PC2 morphospace showed substantial overlap between males and females, although a tendency toward partial separation was apparent, particularly along PC1. Thus, the major axes of overall shape variation were not exclusively sex-dependent, despite the presence of sex-related differences in orbital morphology (Figure 4).

**Figure 4:**
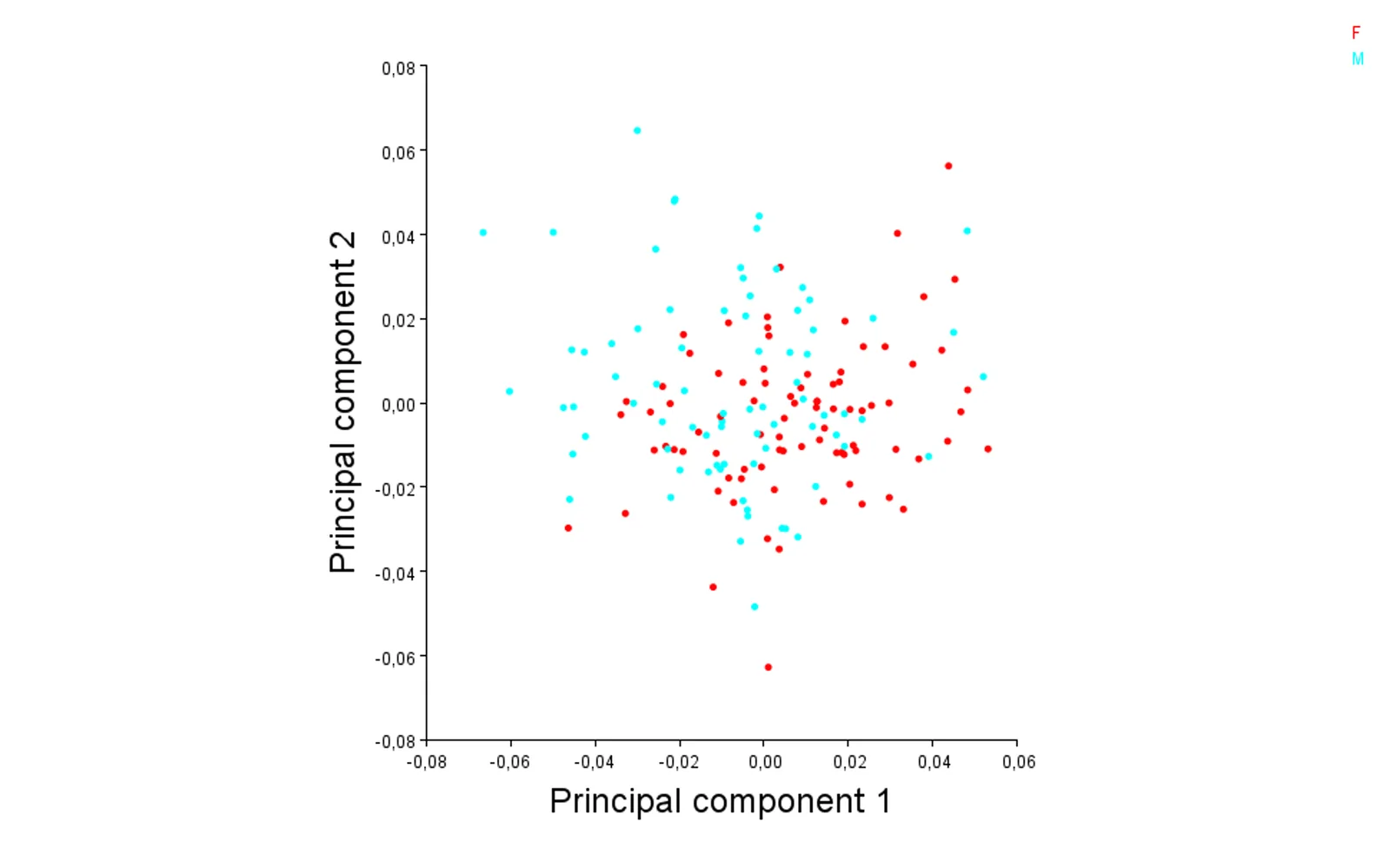
Scatterplot of PC1 and PC2 scores derived from Procrustes-aligned orbital landmark coordinates. PC1 and PC2 accounted for 26.24% and 19.13% of total shape variation, respectively. Male and female configurations showed substantial overlap, with a tendency toward partial separation primarily along PC1. Blue dots represent males, while red dots represent females. PC, principal component

Allometry

A significant relationship was observed between orbital size and shape. Multivariate regression of Procrustes coordinates on log centroid size showed that size accounted for 5.72% of the total shape variation in the complete sample (10,000 permutations, p < 0.001).

Because centroid size differed substantially between males and females, a pooled within-sex regression was performed to evaluate allometry independently of the between-sex size difference. This analysis remained significant, with the log centroid size explaining 4.77% of the within-group shape variation (10,000 permutations, p < 0.001).

Separate regressions performed within each sex also identified significant allometric effects in both groups. In males, centroid size accounted for 3.78% of shape variation (p = 0.0062), whereas in females, it accounted for 7.13% (p < 0.001). These values indicate that size-related shape variation was present in both sexes and was proportionally greater in females.

Discriminant Function Analysis

Discriminant function analysis based on the uncorrected Procrustes shape coordinates demonstrated significant differences between male and female orbital shapes. The Procrustes distance between the group mean configurations was 0.0222, while the Mahalanobis distance was 1.629. Permutation testing confirmed significant differences between the sexes for both Procrustes distance and T² (p < 0.001). The discriminant function for shape correctly classified 130/163 (79.8%) individuals, including 69/85 (81.2%) females and 61/78 (78.2%) males. Following LOOCV, the overall accuracy decreased to 116/163 (71.2%), with 62/85 (72.9%) females and 54/78 (69.2%) males correctly classified.

After correcting for allometry using residuals from the pooled within-sex regression, significant sexual shape differences persisted. The Procrustes distance remained almost unchanged at 0.0220, whereas the Mahalanobis distance increased to 1.927 (p < 0.001). The cross-validated classification accuracy increased to 121/163 (74.2%), with 64/85 (75.3%) females and 57/78 (73.1%) males correctly classified. The minimal change in Procrustes distance before and after allometric correction indicates that the observed sexual difference in orbital shape was not primarily attributable to differences in overall size.

The predictive contribution of size was subsequently evaluated. Centroid size alone achieved an overall LOOCV accuracy of 119/163 (73.0%). When centroid size was combined with the uncorrected shape information, the model showed a cross-validated accuracy of 125/163 (76.7%), with 67/85 (78.8%) females and 58/78 (74.4%) males correctly classified (Table 10).

**Table 10:** Sex classification performance of geometric morphometric models Classification performance is presented as the number of correctly classified individuals/total number of individuals with the corresponding percentage, n/N (%). All classification accuracies were evaluated using leave-one-out cross-validation.

Model	Female, n/N (%)	Male, n/N (%)	Overall, n/N (%)
Uncorrected shape	62/85 (72.9%)	54/78 (69.2%)	116/163 (71.2%)
Allometry-corrected shape	64/85 (75.3%)	57/78 (73.1%)	121/163 (74.2%)
Centroid size	60/85 (70.6%)	59/78 (75.6%)	119/163 (73.0%)
Shape + centroid size	67/85 (78.8%)	58/78 (74.4%)	125/163 (76.7%)

## Discussion

In the present study, linear measurements and geometric morphometric approaches were applied to the orbital region for sex estimation. The linear measurements revealed that sexual dimorphism was expressed predominantly in the transverse dimensions of the orbital region, whereas OBH showed only limited differences between males and females. OBB, IOB, and BOB were all greater in males, with BOB showing the largest absolute difference between the sexes. In contrast, OBH did not differ significantly between males and females for either the right or left orbit. The OI was significantly higher in females bilaterally, a finding that mainly reflects the smaller OBB observed in females in the presence of relatively similar OBHs between the sexes.

The present results on univariate discriminant performance show notable agreement with the previous craniometric study on the Romanian population by Marinescu et al. [14]. In their osteological sample, OBB was significantly greater in males and achieved a cross-validated sex-classification accuracy of 75%, whereas OBH showed substantially weaker discriminatory performance, with an accuracy of only 57%. In the present study, OBB achieved cross-validated accuracies of 114/163 (69.9%) on the right side and 112/163 (68.7%) on the left side, whereas OBH showed lower accuracies of 86/163 (52.8%) and 87/163 (53.4%), respectively. Among all individual linear variables, BOB provided the highest classification accuracy, correctly classifying 119/163 (73.0%) individuals.

Our findings are consistent with those reported in the Croatian population, where BOB was also the best-performing individual measurement, with a cross-validated accuracy of 71.88% [22]. Similar results were reported in the Saudi Arabian population, where BOB achieved an accuracy of 73.5%; however, in that population, right OBH provided the highest classification accuracy, reaching 83.8% [11].

The multivariate discriminant model in the present study correctly classified 121/163 (74.2%) individuals in the original sample, with accuracy decreasing to 114/163 (69.9%) after LOOCV. This performance is comparable to the 73.45% cross-validated accuracy reported for the Croatian population [22] and to the 73.2% multivariate accuracy reported for a Turkish population [23]. Lower performance has been reported in a Greek sample, where a machine-learning model based on orbital measurements achieved an accuracy of approximately 68% [24]. In contrast, substantially higher multivariate accuracies have been reported in Indian samples (approximately 79-92%) [25-27], and the Saudi Arabian population (92.1%) [11].

Geometric morphometric analysis demonstrated that the sexual dimorphism of the orbital region extended beyond differences in conventional linear dimensions. The centroid size was significantly greater in males than in females, with a large effect size, indicating substantial sexual dimorphism in overall orbital size. Shape variation was summarized by PCA, with the first three principal components accounting for 60.42% of the total variation. Nevertheless, considerable overlap between male and female configurations was evident in the PCA morphospace, indicating that the major axes of orbital shape variation were not exclusively related to sex.

Despite this overlap, discriminant analysis confirmed the presence of significant sex-related differences in the 3D orbital shape. The Procrustes distance between the mean shapes of males and females was 0.0222, and the Mahalanobis distance was 1.629, with permutation tests confirming significant group differences (p < 0.001). The shape-based discriminant function correctly classified 130/163 (79.8%) individuals in the original sample, although the accuracy decreased to 116/163 (71.2%) after cross-validation, with 62/85 (72.9%) females and 54/78 (69.2%) males correctly classified.

Orbital shape was also significantly influenced by size, with log centroid size accounting for 5.72% of the total shape variation. After correction for within-sex allometry, significant differences between male and female shapes persisted, while the cross-validated classification accuracy increased slightly to 121/163 (74.2%) individuals. This suggests that sexual shape differences were not simply a consequence of the larger overall orbital size observed in males.

Centroid size alone achieved a cross-validated classification accuracy of 119/163 (73.0%) individuals, which was similar to that obtained from shape alone, with 116/163 (71.2%) individuals correctly classified. However, combining centroid size and shape showed the highest observed accuracy, correctly classifying 125/163 (76.7%) individuals. These findings indicate that orbital size and shape provide partially complementary information for sex estimation, with their combination showing a higher cross-validated accuracy than either component considered independently.

Previous geometric morphometric studies of the orbital region have reported variable classification performances across Europe. In a Bosnian sample, shape-based classification correctly classified 82.01% of males and 80.55% of females [12]. A study conducted in a Central European population reported an overall orbital shape classification accuracy of approximately 74% [28]. In a contemporary French sample, the original discriminant analysis correctly classified 90.9% of males and 86.4% of females; however, performance decreased substantially to 68.1% following LOOCV, emphasizing the importance of internal validation when evaluating discriminatory performance [13]. In a Greek population, orbital shape correctly classified 74.4% of males and 70.4% of females [29], whereas a Bulgarian study reported a cross-validated accuracy of 68.8% for orbital shape [30].

Overall, the wide variation in classification performance across populations using the linear and geometric approaches reinforces the population-specific expression of orbital sexual dimorphism.

The cross-validated accuracies of the linear, centroid size, and shape models were broadly similar when these approaches were considered independently. The combined shape-and-size model showed the highest observed cross-validated accuracy, suggesting that orbital size and shape may provide complementary information for sex estimation.

Sex-specific classification performance showed modest asymmetry in the shape-based models, with consistently higher accuracy among females. Cross-validated shape classification correctly classified 62/85 (72.9%) females and 54/78 (69.2%) males, while the corresponding accuracies after allometric correction were 64/85 (75.3%) in females and 57/78 (73.1%) in males. A similar pattern was observed for the combined shape and size model, which correctly classified 67/85 (78.8%) females and 58/78 (74.4%) males. Because equal prior probabilities were used for both sexes, this asymmetry cannot be attributed to unequal classification priors. Instead, it may reflect differences in the degree of overlap between male and female morphometric distributions, with a slightly greater proportion of male configurations occupying regions of shape space shared with females.

Several limitations should be acknowledged. Although LOOCV provided internal validation, no independent external validation sample was available; therefore, some model optimism may remain, and the generalizability of the reported accuracies requires confirmation in independent samples. Because the sample was derived from a retrospective medico-legal case series rather than from population-based sampling, it may not be fully representative of the general Romanian adult population. In addition, height and body weight were not systematically available and could not be evaluated as potential confounding factors. Orbital geometry was represented by 10 discrete anatomical landmarks, which may not fully characterize the continuous contour of the orbital region. Finally, although the female and male samples were slightly unequal in size, equal prior probabilities were used in discriminant classification, reducing the potential influence of group prevalence on classification outcomes.

## Conclusions

The orbital region showed significant sexual dimorphism in the contemporary Romanian sample, particularly in transverse dimensions, whereas OBH displayed limited discriminatory value. Geometric morphometric analysis further demonstrated significant sex-related differences in both centroid size and orbital shape. Conventional linear measurements showed lower cross-validated classification accuracy than the combined centroid size and shape model. The combined centroid size and shape model showed the highest observed cross-validated classification accuracy, correctly classifying 125/163 (76.7%) individuals. These findings suggest that when 3D size and shape information are considered together, they may provide useful complementary evidence for sex estimation with potential indirect relevance to forensic facial reconstruction.
